# Glucose-6-phosphate dehydrogenase deficiency among Yemeni children residing in malaria-endemic areas of Hodeidah governorate and evaluation of a rapid diagnostic test for its detection

**DOI:** 10.1186/s12936-016-1372-9

**Published:** 2016-06-21

**Authors:** Rashad Abdul-Ghani, Mohammed A. K. Mahdy, Reyadh Saif-Ali, Sameer A. Alkubati, Abdulhabib R. Alqubaty, Abdullah A. Al-Mikhlafy, Samira M. Al-Eryani, Abdusalam M. Al-Mekhlafi, Ali Alhaj

**Affiliations:** Tropical Disease Research Center, Faculty of Medicine and Health Sciences, University of Science and Technology, Sana’a, Yemen; Department of Parasitology, Faculty of Medicine and Health Sciences, Sana’a University, Sana’a, Yemen; Department of Biochemistry, Faculty of Medicine and Health Sciences, Sana’a University, Sana’a, Yemen; Department of Critical Care Nursing, Faculty of Medicine and Health Sciences, Hodeidah University, Hodeidah, Yemen; Department of Biochemistry, Faculty of Medicine and Health Sciences, University of Science and Technology, Sana’a, Yemen; Department of Community Medicine, Faculty of Medicine and Health Sciences, University of Science and Technology, Sana’a, Yemen

**Keywords:** Glucose-6-phosphate dehydrogenase, Malaria, Rapid diagnostic test, Primaquine, Yemen

## Abstract

**Background:**

Glucose-6-phosphate dehydrogenase (G6PD) deficiency, the most common genetic enzymopathy worldwide, is associated with an acute haemolytic anaemia in individuals exposed to primaquine. The present study aimed to determine G6PD deficiency among Yemeni children in malaria-endemic areas as well as to assess the performance of the CareStart™ G6PD rapid diagnostic test (RDT) for its detection.

**Methods:**

A cross-sectional study recruiting 400 children from two rural districts in Hodeidah governorate was conducted. Socio-demographic data and blood samples were collected and G6PD deficiency was qualitatively detected in fresh blood in the field using the CareStart™ G6PD RDT, while the enzymatic assay was used to quantitatively measure enzyme activity. Performance of the CareStart™ G6PD RDT was assessed by calculating its sensitivity, specificity, negative predictive value (NPV), and positive predictive value (PPV) against the reference enzymatic assay.

**Results:**

The ranges of enzyme activity were 0.14–18.45 and 0.21–15.94 units/g haemoglobin (U/gHb) for males and females, respectively. However, adjusted male median G6PD activity was 5.0 U/gHb. Considering the adjusted male median as representing 100 % normal enzyme activity, the prevalence rates of G6PD deficiency were 12.0 and 2.3 % at the cut-off activities of ≤60 and ≤10 %, respectively. Multivariable analysis showed that gender, district of residence and consanguinity between parents were independent risk factors for G6PD deficiency at the cut-off activity of ≤30 % of normal. The CareStart™ G6PD RDT showed 100 % sensitivity and NPV for detecting G6PD deficiency at the cut-off activities of ≤10 and ≤20 % of normal activity compared to the reference enzymatic method. However, it showed specificity levels of 90.0 and 95.4 % as well as positive/deficient predictive values (PPVs) of 18.0 and 66.0 % at the cut-off activities of ≤10 and ≤20 %, respectively, compared to the reference method.

**Conclusions:**

G6PD deficiency with enzyme activity of ≤60 % of normal is prevalent among 12.0 % of children residing in malaria-endemic areas of Hodeidah governorate, with 2.3 % having severe G6PD deficiency. Gender, district of residence and consanguinity between parents are significant independent predictors of G6PD deficiency at the cut-off activity of ≤30 % of normal among children in malaria-endemic areas of Hodeidah. The CareStart™ G6PD RDT proved reliable as a point-of-care test to screen for severely G6PD-deficient patients, with 100 % sensitivity and NPV, and it can be used for making clinical decisions prior to the administration of primaquine in malaria elimination strategies.

## Background

Malaria elimination from low and moderate endemic countries mainly depends on vector control and prompt diagnosis and treatment of infected patients [1]. With absence of clinically proven vaccines to block malaria transmission to humans [2, 3], there is an increasing interest in blocking malaria transmission to mosquitoes through the strategic use of gametocytocidal anti-malarial drugs [4]. Although artemisinin derivatives are effective in treating uncomplicated symptomatic falciparum malaria and reducing disease transmission by targeting young gametocytes of *Plasmodium falciparum* [5–8], they show little or no activity against *P. falciparum* mature gametocytes [9]. Therefore, the effect of artemisinin-based combination therapy (ACT) on the transmission of *P. falciparum* is only moderate based on field data [10]. The 8-aminoquinoline, primaquine is the only drug commonly used to kill mature *P. falciparum* gametocytes and to clear sub-microscopic gametocytaemia after treatment with other anti-malarial drugs [11, 12]. Despite being recommended by the World Health Organization (WHO) for blocking *P. falciparum* transmission and for preventing *Plasmodium vivax* relapses, primaquine has not been used widely in malaria-endemic areas due to concerns about causing acute haemolytic anaemia in patients with glucose-6-phosphate dehydrogenase (G6PD) deficiency [13].

G6PD deficiency is an X-linked recessive hereditary enzymopathy affecting millions of people worldwide and is more common in malaria-endemic countries [13–15]. Haemolysis as a result of G6PD deficiency most commonly affects haemizygous males compared to homozygous females, while it depends on the balance between the mixed G6PD-normal and -deficient populations of red cells in heterozygous females [16]. Several methods have been developed for the detection of G6PD deficiency, including the qualitative fluorescent spot test (FST), brilliant cresyl blue dye test and the quantitative enzymatic assays [14]. Although the International Committee for Standardization in Haematology (ICSH) recommends the FST for determining G6PD deficiency [17], the quantitative G6PD enzymatic assay remains the reference method [18–20]. With the current trend of developing point-of-care diagnostics, rapid diagnostic tests (RDTs) have been developed and evaluated for screening of G6PD deficiency in the field [21–23].

In Yemen, malaria is a major public health problem, with more than 40 % of the population being at high risk and more than 100,000 microscopy- and RDT-confirmed cases being reported in 2013 [24]. Although real estimates of malaria prevalence in the study area are not available, partly because of the current social unrest in the country, Al-Mekhlafi et al. [25] reported a prevalence rate of 15.3 % of falciparum malaria among febrile patients in Hodeidah in 2009. However, the situation might be even worse if asymptomatic and sub-microscopic infections were considered.

Recently, a G6PD deficiency prevalence rate of 7.1 % (36/508) has been reported among male blood donors in Sana’a city [26]. However, the prevalence of G6PD deficiency in malaria-endemic areas in Yemen remains unclear. The national malaria drug policy in Yemen adopts primaquine in combination with chloroquine for treating vivax malaria and recommends a 14-day primaquine dosage for its radical cure [27]. Moreover, primaquine-based transmission-blocking strategies for paving the way for elimination of falciparum malaria are yet to be adopted in the country [28]. Therefore, there is a need to include primaquine in the anti-malarial policy as a measure to prevent the spread of falciparum malaria through targeting mature gametocytes of the parasite as a transmission-blocking strategy. However, such a measure is limited by the prevalence and severity of G6PD deficiency in malaria-endemic areas, as well as the absence of easy-to-use diagnostics for its detection. In pursuit of malaria elimination, there is an urgent need to evaluate the performance of RDTs for screening of patients with severe G6PD deficiency prior to primaquine use in malaria-endemic areas. Although primaquine is recommended as a single dose of 0.25 mg with ACT to patients with falciparum malaria in low transmission settings without prior testing, its safety among pregnant women and infants less than 6 months is yet to be elucidated [29]. Moreover, data about safety and efficacy of the WHO recommendation for the use of a single low-dose primaquine approach are still limited [30]. For this reason, G6PD testing is critically needed before primaquine use in a high-dose regimen for the radical cure of vivax malaria as well as before its use as a gametocytocide for falciparum malaria in certain circumstances. This is particularly important with the documented evidence of acute haemolysis caused by primaquine among Yemeni patients. In this context, Abdullah [31] reported acute intravascular haemolysis in 57 cases after a single primaquine administration of 0.75 mg with artesunate, which was associated with moderate to severe anaemia, hyperbilirubinaemia and death as a result of acute renal failure following massive haemolysis.

Therefore, the aim of the present study was to determine the prevalence and severity of G6PD deficiency among children residing in malaria-endemic areas of Hodeidah governorate and to evaluate the performance of the CareStart™ G6PD RDT for screening of G6PD deficiency compared to the reference enzymatic method.

## Methods

### Study design, setting and ethical clearance

**Fig. 1:**
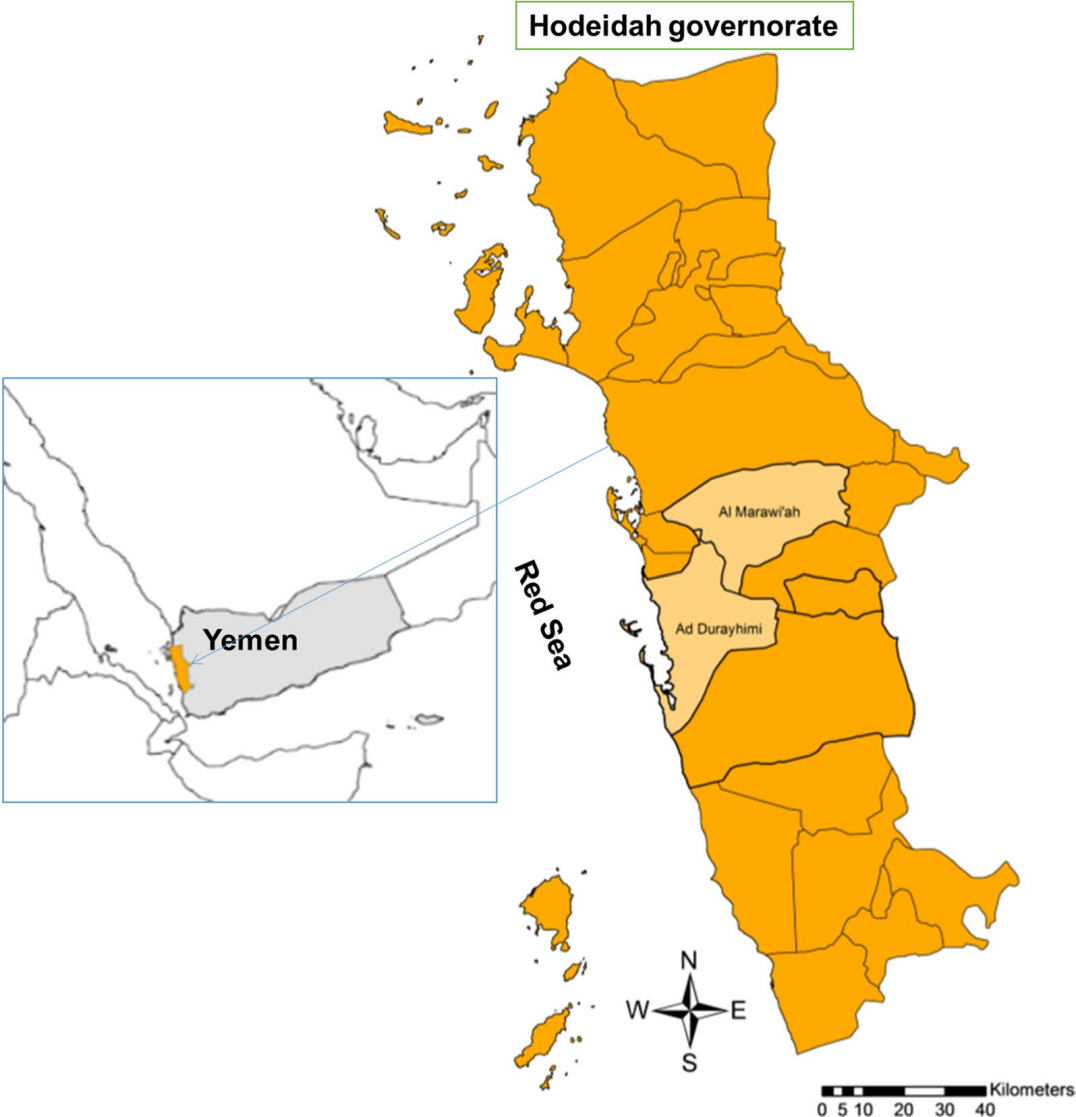
Map of Yemen showing Hodeidah governorate and the location of the two study districts

A cross-sectional study design was adopted to determine the prevalence and severity of G6PD deficiency among children residing in malaria-endemic areas of Hodeidah governorate during March 2016. Hodeidah is a coastal governorate and port on the Red Sea at the coordinates of 14°48′08″N 42°57′04″E (Fig. 1). According to the latest population census, its total population is about 2,279,000 with 1,556,000 being in rural areas and 723,000 in urban areas [32]. The study protocol was approved by the Ethics Committee of the University of Science and Technology, Sana’a. In addition, informed consent was obtained from children’s parents or guardians after clearly explaining the study objectives.

### Sampling strategy

Sample size was calculated according to the WHO practical manual for the determination of sample size in health studies [33], at a confidence level of 95 % and an expected G6PD deficiency prevalence of 50 % because no data are available from malaria-endemic areas in the country. Applying the above criteria, the minimum sample size required was 384 children. However, 400 children were recruited in the present study.

The malaria-endemic districts of Hodeidah were listed, and two districts were then randomly selected taking into consideration the safe and easy access; namely, Ad Durayhimi and Al Marawi’ah districts (Fig. 1). This was followed by a two-stage, random sampling of villages and households from each district. All children with age from 2 to 15 years in the selected households were invited to voluntarily participate, where the number of children recruited from each district was proportional to the total population of children in the district.

### Blood sampling, data collection and laboratory investigations

Three millilitres of venous blood were collected into pre-labelled EDTA tubes from each participant, kept in an icebox and transported on the same day to the laboratory of the Military Hospital in Hodeidah city. Data on gender, age, residence, and consanguinity between parents were collected using a pre-designed data collection sheet. Haemoglobin (Hb) concentration was measured using Sysmex KX-21N™ Automated Haematology Analyser (Sysmex Corp, Chuo-Ku, Kobe, Japan).

Qualitative screening for G6PD deficiency was performed on fresh venous blood in the field using CareStart™ G6PD RDT (AccessBio, New Jersey, USA) according to the manufacturer’s instructions. G6PD activity was quantitatively measured within 14 h of blood collection using Randox G-6-PDH kits, Cat No PD2616 (Randox Laboratories Ltd, Antrim, UK) according to the manufacturer’s instructions. The technician who carried out the quantitative enzymatic assay was blinded to the results of the CareStart™ G6PD DT.

### Data analysis

Data were entered and analysed using the IBM SPSS Statistics version 21.0 for Windows (IBM Corp., Armonk, NY, USA). Categorical variables were presented as proportions, and differences and associations were tested using Pearson’s Chi square test. The odds ratio (OR) and its corresponding 95 % confidence interval (CI) were reported in the univariate analysis of predictors of G6PD deficiency, and multivariable analysis using a logistic regression model was used to determine independent predictors of deficiency. The performance of CareStart™ G6PD RDT was determined by calculating its sensitivity, specificity, negative/normal predictive value (NPV), and positive/deficient predictive value (PPV) against the reference enzymatic assay. The agreement between CareStart™ G6PD RDT and the reference method was tested using Cohen’s Kappa coefficient (*Kc*) [34]. The following scale was used to determine the strength of agreement between the two tests: slight: *Kc* = 0.01–0.20; fair: *Kc* = 0.21–0.40; moderate: *Kc* = 0.41–0.60; substantial: *Kc* = 0.61–0.8; or almost perfect: *Kc* = 0.81–1 [35]. The significance was considered at p < 0.05.

## Results

### Characteristics of the study population and determination of G6PD-enzymatic activity

Of the 400 children enrolled in the present study, 55 % were males and 45 % were females, with a median age of eight years [interquartile range (IQR) 6–11]. The median Hb concentration of the children was 11.0 g/dL (IQR 9.9-11.7). About two-thirds of children were residing in Al Marawi’ah district; however, consanguinity between children’s parents was higher in Ad Durayhimi district than in Al Marawi’ah district, being 46.8 and 35.1 %, respectively (Table 1). Table 2 shows the G6PD activity reference values among the studied children as determined by the quantitative reference enzymatic method. The median G6PD activity was equal for both female and male children; however, the ranges of enzyme activity were 0.14–18.45 U/gHb and 0.21–15.94 U/gHb, respectively. The adjusted male median G6PD activity was 5.0 U/gHb (IQR = 4.3–5.7 U/gHb), where it was used to represent 100 % G6PD activity among the study population.

**Table 1:** Characteristics of the study population

Characteristic	
Median age in years (IQR)	8 (6–11)
Gender	
Male *n* (%)	219 (55)
Female *n* (%)	181 (45)
District of residence	
Al Marawi’ah *n* (%)	259 (65)
Ad Durayhimi *n* (%)	141 (35)
Median Hb in g/dL (IQR)	11 (9.9–11.7)
Consanguinity between parents (by district)	
Al Marawi’ah *n* (%)	91/259 (35.1)
Ad Durayhimi *n* (%)	66/141 (46.8)

Total number of children enrolled in the present study is 400

*Hb* haemoglobin, *IQR* interquartile range

**Table 2:** G6PD activity among the study population by the reference enzymatic method

Reference values of G6PD activity (U/gHb)	Female (*n* = 181)	Male (*n* = 219)	Adjusted male (*n* = 194)^a^
Range	0.14–18.45	0.21–15.94	1.23–15.94
Median (IQR)	4.7 (4.1–5.5)	4.7 (3.9–5.7)	5.0 (4.3–5.7)

*G6PD* glucose-6-phosphate dehydrogenase, *U/gHb* units per gram haemoglobin, *SD* standard deviation, *IQR* interquartile range

^a^Reference values were re-calculated after excluding severe G6PD deficiency, and the adjusted male median G6PD activity was used to represent 100 % enzyme activity

### Prevalence and distribution of G6PD deficiency among children in Hodeidah governorate

Considering that the adjusted male median G6PD activity (5.0 U/gHb) represents normal (100 %) enzyme activity, cut-off values representing ≤10, ≤20, ≤30, ≤40, ≤50 and ≤60 % of the normal G6PD activity were determined. The prevalence rates of G6PD deficiency ranged from 2.3 % for those with ≤10 % of normal activity to 12.0 % for those with ≤60 % of normal activity (Table 3). Male children showed higher G6PD deficiency prevalence rates than females at all cut-off values; however, significant differences were found at the cut-offs of ≤20–40 % of normal activity. In addition, G6PD deficiency was significantly more prevalent among children residing in Ad Durayhimi compared to those from Al Marawi’ah and among children with consanguineous compared to those with non-consanguineous parents at all cut-off activities (Table 3).

**Table 3:** Prevalence of G6PD deficiency among children in Hodeidah according to the cut-off activities used to determine the enzyme deficiency

G6PD activity cut-off^a^	*N*	Prevalence of G6PD deficiency % (95 % CI)					
		≤10 % (≤0.5 U/gHb)	≤20 % (≤1.0 U/gHb)	≤30 % (≤1.5 U/gHb)	≤40 % (≤2.0 U/gHb)	≤50 % (≤2.5 U/gHb)	≤60 % (≤3.0 U/gHb)
Overall	400	2.3 (1–4)	8.3 (6–11)	9.5 (7–13)	10.3 (8–14)	11.3 (9–15)	12.0 (9–16)
Gender							
Male	219	2.7 (1–6)**	11.4 (8–16)	12.8 (9–18)	13.2 (9–18)	13.7 (10–19)**	14.2 (10–19)**
Female	181	1.7 (1–5)	4.4 (2–8)	5.5 (3–10)	6.6 (4–11)	8.3 (5–13)	9.4 (6–15)
District of residence							
Al Marawi’ah	259	1.2 (0–3)	5.4 (3–9)	6.6 (4–10)	7.7 (5–12)	8.9 (6–13)	9.3 (6–13)
Ad Durayhimi	141	4.3 (2–9)^#^	13.0 (9–20)	14.9 (10–22)	14.9 (10–22)	16.6 (11–22)	17.0 (12–24)
Consanguinity between parents							
Yes	157	4.5 (2–9)	13.4 (9–20)	14.0 (9–20)	14.0 (9–20)^#^	15.9 (11–22)	17.2 (12–24)
No	243	0.8 (0–3)	4.9 (3–8)	6.6 (4–10)	7.8 (5–12)	8.2 (5–12)	8.6 (6–13)

*G6PD* glucose-6-phosphate dehydrogenase, *CI* confidence interval, *U/gHb* units per gram haemoglobin

** p > 0.05; ^#^ p = 0.05; p < 0.05 for all other differences

^a^Calculated from the median value of 5.0 that is equivalent to 100 % activity

### Predictors of G6PD deficiency among children in Hodeidah

Considering that the cut-off of ≤30 % of normal activity as the threshold for determining enzyme deficiency before primaquine administration, univariate analysis showed that gender is significantly associated with G6PD deficiency, where male children were at a 2.5-fold higher risk of G6PD deficiency than females (OR  =  2.5; 95 % CI 1.18–5.31, p = 0.014). In addition, the district of residence (OR  =  2.5; 95 % CI 1.27–4.89, p = 0.007) and consanguinity between the parents (OR  =  2.3; 95 % CI 1.17–4.56, p = 0.013) were significant predictors of G6PD deficiency among children residing in malaria-endemic areas of Hodeidah. Children residing in Ad Durayhimi and whose parents are consanguineous were at a higher risk of developing G6PD deficiency compared to those residing in Al Marawi’ah and whose parents are non-consanguineous (Table 4). Moreover, multivariable analysis identified gender, district of residence and consanguinity between parents as independent risk factors for G6PD deficiency at the cut-off of ≤30 % of normal activity among children in Hodeidah.

**Table 4:** Predictors of G6PD deficiency among children in Hodeidah according to the cut-off activities used to determine the enzyme deficiency

Variable^b^	G6PD cut-off activity of ≤30 % (≤1.5 U/gHb)^a^			
	N	*n* (%)	OR (95 % CI)	p value
Gender				
Male	219	28 (12.8)	2.5 (1.18–5.31)	0.014
Female	181	10 (5.5)	Reference	
District of residence				
Ad Durayhimi	141	21 (14.9)	2.5 (1.27–4.89)	0.007
Al Marawi’ah	259	17 (6.6)	Reference	
Consanguinity between parents				
Yes	157	22 (14.0)	2.3 (1.17–4.56)	0.013
No	243	16 (6.6)	Reference	

*OR* odds ratio, *CI* confidence interval, *U/gHb* units per gram haemoglobin

^a^Calculated from the median value of 5.0 that is equivalent to 100 % activity

^b^All variables were confirmed as independent risk factors using stepwise forward logistic regression

### Diagnostic performance of the CareStart™ G6PD RDT for detecting G6PD deficiency among children in Hodeidah

At cut-off activities of ≤10 and ≤20 % of normal G6PD activity, the CareStart™ G6PD RDT showed 100 % sensitivity (95 % CI 70–100 and 90–100 %, respectively) and 100 % NPV (95 % CI 99–100 %, each) compared to the reference method. However, it had specificity levels of 90 % (95 % CI 86–92 %) and 95.4 % (95 % CI 93–97 %) as well as PPVs of 18 % (95 % CI 10–31 %) and 66 % (95 % CI 52–78 %) at the cut-off activities of ≤10 and ≤20 %, respectively, compared to the reference method. The CareStart™ G6PD RDT showed a fair degree of agreement (89.8 %; *Kc* = 0.278, p < 0.001) and a substantial degree of agreement (95.8 %; *Kc* = 0.773, p < 0.001) with the reference method in detecting G6PD deficiency at cut-off values of ≤10 and ≤20 % of normal activity, respectively (Table 5).

**Table 5:** Performance of the CareStart™ G6PD RDT for detecting G6PD deficiency in comparison to the reference enzymatic method

	G6PD activity cut-off values^a^					
	≤10 % (≤0.5 U/gHb)	≤20 % (≤1.0 U/gHb)	≤30 % (≤1.5 U/gHb)	≤40 % (≤2.0 U/gHb)	≤50 % (≤2.5 U/gHb)	≤60 % (≤3.0 U/gHb)
RDT-deficient vs EM-deficient (*n*)	9	33	37	39	39	39
RDT-deficient vs EM-normal (*n*)	41	17	13	11	11	11
RDT-normal vs EM-normal (*n*)	350	350	349	348	344	341
RDT-normal vs EM-deficient (*n*)	0	0	1	2	6	9
Sensitivity % (95 % CI)	100 (70–100)	100 (90–100)	97.4 (87–100)	95.1 (84–99)	86.7 (74–94)	81.3 (68–90)
Specificity % (95 % CI)	90.0 (86–92)	95.4 (93–97)	96.4 (94–98)	96.9 (94–98)	96.9 (95–98)	96.9 (94–98)
PPV % (95 % CI)	18.0 (10–31)	66.0 (52–78)	74.0 (60–84)	78.0 (65–87)	78.0 (65–87)	78.0 (65–87)
NPV % (95 % CI)	100 (99–100)	100 (99–100)	99.7 (98–100)	99.4 (98–100)	98.3 (96–99)	97.4 (95–99)
% Agreement (*Kc*)^b^	89.8 (0.278)	95.8 (0.773)	96.6 (0.822)	96.8 (0.839)	95.8 (0.797)	95.1 (0.767)

*G6PD* glucose-6-phosphate dehydrogenase, *RDT* rapid diagnostic test, *EM* enzymatic method, *CI* confidence interval, *PPV* positive (deficient) predictive value, *NPV* negative (normal) predictive value, *U/gHb* units per gram haemoglobin, *Kc* Cohen’s kappa coefficient

^a^Calculated from the median value of 5.0 that is equivalent to 100 % activity

^b^% Agreement was calculated by the summation of the number of deficient and normal cases by both RDT and EM divided by the total number of cases and was found significant between RDT and EM at all cut-off activities with p < 0.001

On the other hand, the sensitivity of the CareStart™ G6PD RDT ranged from 81.3 % (95 % CI 68–90 %) to 97.4 % (95 % CI 78–100 %) for detecting G6PD deficiency at the cut-offs of ≤60 and ≤30 %, respectively, compared to the reference method, with comparable specificity levels of 96.4–96.9 %. In addition, comparable PPVs and NPVs of 74–78 and 97.4–99.7 %, respectively, were found for detecting G6PD deficiency using the CareStart™ G6PD RDT compared to the reference method at the cut-off activities of ≤30–60 % of normal G6PD activity. The degrees of agreement between the CareStart™ G6PD RDT were almost perfect at the cut-off activities of ≤30 and ≤40 %, being 96.6 %; *Kc* = 0.822 and 96.8 %; *Kc* = 0.839, respectively. However, a substantial degree of agreement was found at cut-off activities of ≤50 and ≤60 %, being 95.8 %; *Kc* = 0.797 and 95.1 %; *Kc* = 0.767, respectively (Table 5).

## Discussion

This is the first study to determine the prevalence of G6PD deficiency in malaria-endemic areas of Yemen and to evaluate a point-of-care diagnostic tool for its detection. Because the normal G6PD reference range has not been established in the country yet, the adjusted male median of G6PD activity (5.0 U/gHb) was considered as representing 100 % normal enzyme activity. Given that an internationally accepted cut-off for G6PD deficiency is yet to be established, G6PD deficiency cut-off thresholds were determined as ranging from ≤10 to 60 % of the adjusted male median according to the WHO classification, with those having ≤10 % of normal enzyme activity were considered severely G6PD-deficient [36].

The overall prevalence of G6PD deficiency was 12.0 % for children with ≤60 % normal activity (≤3.0 U/gHb). However, the prevalence of severe G6PD deficiency (≤10 %; ≤0.5 U/gHb) was 2.3 %. This is in contrast to a recent finding by Al-Nood et al. [26], who reported that all, except for one, of 7.1 % asymptomatic male blood donors attending the Blood Bank Department of the National Centre of the Public Health Laboratories in Sana’a were severely G6PD-deficient, having a G6PD activity of <10 % of normal. In addition, the G6PD deficiency prevalence rate is lower than the rates in malaria-endemic areas in sub-Saharan Africa, where rates as high as 30 % have been reported [13].

In general, 10–30 % of normal G6PD activity has been predominantly accepted to define severely G6PD-deficient individuals who should be excluded from primaquine administration [20]. In the present study, at the G6PD cut-off-activity of ≤30 % of normal, gender of children was a significant independent predictor for G6PD deficiency. Male children were at a 2.5-fold higher risk than females for developing G6PD deficiency (12.8 vs 5.5 %) in malaria-endemic areas of Hodeidah. This finding is consistent with that recently reported among G6PD-deficient Ethiopian malaria suspects [37]. The higher frequency of G6PD deficiency among males has been reported from different parts of the world and is explained by the fact that G6PD deficiency is an X-linked hereditary disorder [13, 14, 21, 38–40].

In the present study, district of residence and consanguinity between parents were predictors of G6PD deficiency among children residing in malaria-endemic areas of Hodeidah. Residing in Ad Durayhimi and having consanguineous parents predict more than a twofold higher risk for developing G6PD deficiency among children in Hodeidah compared to those living in Al Marawi’ah and having non-consanguineous parents. This is supported by the higher rate of consanguinity between parents in Ad Durayhimi than Al Marawi’ah (46.8 vs 35.1 %). High consanguinity rate within communities is a major determinant of the prevalence and burden of X-linked genetic disorders, including G6PD deficiency [41, 42]. Like gender of the children, multivariable analysis identifies the district of residence and consanguinity as independent risk factors for G6PD deficiency at the cut-off of ≤30 % of normal enzyme activity among children in Hodeidah. Therefore, it is impossible to generalize the G6PD deficiency prevalence rate among children in the present study to other malaria-endemic areas in the country. By the same token, the Yemeni community is characterized by the different traditions related to the preference of consanguinity due to its social heterogeneity and tribal diversity. It is noteworthy that different prevalence and distribution patterns of G6PD variants have been reported from different malaria-endemic areas in the world [13, 43, 44].

Identifying those at high risk of haemolysis induced by primaquine in malaria-endemic areas in limited-resource countries necessitates the evaluation of suitable point-of-care screening tools for G6PD deficiency to serve malaria elimination strategies. Of particular importance is the screening for G6PD deficiency before the use of a high-dose primaquine regimen for the radical cure of vivax malaria. In addition, screening for the enzyme deficiency before low-dose primaquine administration as a gametocytocide with ACT in transmission-blocking strategies is needed for population categories with unclear primaquine safety profile such as pregnant women and under-six-month infants [29]. Although the FST has been recommended by the ICSH as the most appropriate method for qualitative screening in the field, it requires the use of an ultraviolet lamp, water bath and a micropipette for performing the test and a cold chain for the preservation of its reagents [17], making it unsuitable for rural malaria-endemic areas in resource-limited countries. Moreover, its detection threshold has been estimated to be around 20 % of normal enzyme activity in a large-scale study of over 1.2 million newborns [45].

Alternatively, the findings of the present study show the good performance of CareStart™ G6PD RDT for detecting severe G6PD deficiency compared to the reference method among children in rural, malaria-endemic areas of Hodeidah governorate. The ability of the CareStart™ G6PD RDT to detect all severely G6PD-deficient patients is strongly supported by the perfect (100 %) sensitivity and absolute negative predictability in detecting G6PD deficiency and excluding falsely normal G6PD activity in individuals testing negative. It is noteworthy that as the cut-off activity increases, the sensitivity of the CareStart™ G6PD RDT in detecting enzyme deficiency decreases. Nevertheless, the CareStart™ G6PD RDT correctly identifies those with severe deficiency, which makes it reliable in identifying patients at particular risk of acute haemolysis induced by primaquine. This is a practical option because it is difficult to determine the threshold of enzymatic activity that should be set for the ideal RDT to accurately detect G6PD deficiency [46]. These findings correlate with those concluded by two recent studies assessing the performance of the CareStart™ G6PD RDT in screening G6PD deficiency in Thailand [46] and Cambodia [47], where 100 % sensitivity and NPV were found for the detection of <30 % G6PD activity against the quantitative G6PD assay. Adu-Gyasi et al. [48] also reported 100 % sensitivity of the CareStart™ G6PD RDT for detecting G6PD deficiency from capillary blood of Ghanaian participants against Trinity Biotech quantitative G6PD assay; however, a cut-off activity of 75 % (equivalent to 4.1 U/gHb) was adopted for evaluation. In contrast to the findings of the present study, the CareStart™ G6PD RDT sensitivity of 90.0 and 84.8 % were estimated for detecting severely G6PD-deficient Haitian individuals with <10 and <30 % activities of normal, respectively, against Trinity Biotech quantitative assay [49]. Kim et al. [21] also reported lower CareStart™ G6PD RDT sensitivity of 68 % but higher specificity of 100 % for detecting G6PD deficiency in a field evaluation of the test among Cambodians at the lower limit of normal activity (a cut-off activity of 3.6 U/gHb) against the enzymatic assay. Although the sensitivity of the CareStart™ G6PD RDT declines at cut-offs of ≤50 and ≤60 % of normal G6PD activity, its high specificity and NPV at these cut-offs make it helpful in excluding deficiency in those testing negative. Therefore, with the exception of those patients testing positive by the RDT, who may need careful clinical history taking and/or confirmatory testing for G6PD deficiency, primaquine could be given on the basis of the RDT result.

Beyond the good performance in detecting severe G6PD deficiency, the CareStart™ G6PD RDT maintains high sensitivity and specificity of more than 95 % with an almost perfect agreement with the reference method at cut-offs of ≤30 and ≤40 % of normal G6PD activity. On the other hand, the sensitivity of CareStart™ G6PD RDT declines with increasing cut-off activities of normal to reach as low as about 80 % for detecting ≤60 % enzyme activity. However, these cut-off activities are above the threshold recommended to consider when administering primaquine [20]. In addition to its perfect sensitivity, the CareStart™ G6PD RDT also exhibits high specificity of no less than 90 % for detecting severe G6PD deficiency. The field utility of the CareStart™ G6PD RDT for screening of G6PD deficiency is supported by the finding of a previous study that reported its higher sensitivity compared to the FST when using capillary blood [46], making it more appropriate for field screening. Although the CareStart™ G6PD RDT had been reported to show a high rate of as much as 10 % of invalid results when using capillary blood [46], none were observed during the present study. It is noteworthy, however, that Espino et al. [50] reported that the CareStart™ G6PD RDT is more sensitive in detecting G6PD deficiency using venous blood compared to capillary blood in a recent study in the Philippines (93.8 vs 68.8 %, respectively) at a cut-off activity of 30 % of normal. Therefore, further studies are needed to explore the best performance conditions of the CareStart™ G6PD RDT for detecting G6PD deficiency among Yemeni people.

The pivotal role of primaquine in the context of malaria elimination lies in its dual ability as a hypnozoitocide to radically cure *P. vivax* malaria and as a gametocytocide to eradicate mature *P. falciparum* gametocytes [51, 52]. In addition, primaquine is a major component of artemisinin resistance containment programmes to counteract the spread of resistant strains of *P. falciparum* [53]. However, the most life-threatening, primaquine-induced haemolysis occurs among those with the lowest residual G6PD activity [54]. Therefore, the ability of the CareStart™ G6PD RDT to reliably detect all severely G6PD-deficient cases adequately addresses the minimum criteria to be adopted as a screening RDT for severe G6PD deficiency among Yemenis residing in malaria-endemic areas before administering primaquine as part of future malaria elimination strategies. Moreover, the long-term stability and conserved performance of the CareStart™ G6PD RDT at high temperatures [21] makes it appropriate for use in malaria-endemic areas in Yemen.

## Conclusions

The prevalence of G6PD with ≤60 % of normal activity among children residing in malaria-endemic areas of Hodeidah governorate is 12.0 %, with about 2.3 % having severe G6PD deficiency of ≤10 % of normal activity. Gender, district of residence and consanguinity between parents are significant independent predictors of G6PD deficiency at the cut-off of ≤60 % of normal enzyme activity among children in malaria-endemic areas of Hodeidah. Male children who are residing in Ad Durayhimi and whose parents are consanguineous are a higher risk in developing G6PD deficiency at the cut-off of ≤30 % of normal enzyme activity. The CareStart™ G6PD RDT proved reliable as a point-of-care test to screen for severely G6PD-deficient patients and to make clinical decisions prior to primaquine administration in malaria elimination strategies. It shows 100 % sensitivity and NPV for detecting severe G6PD deficiency against the reference method. It is recommended that the performance of the CareStart™ G6PD RDT is also evaluated for the detection of G6PD deficiency among malaria-infected patients compared to normal controls. In addition, genotyping studies for the exploration of G6PD variants should be conducted in malaria-endemic areas of Yemen.
